# Development of Optical Fiber Based Measurement System for the Verification of Entrance Dose Map in Pencil Beam Scanning Proton Beam

**DOI:** 10.3390/s18010227

**Published:** 2018-01-15

**Authors:** Jaeman Son, Se Byeong Lee, Youngkyung Lim, Sung Yong Park, Kwanho Cho, Myonggeun Yoon, Dongho Shin

**Affiliations:** 1 Department of Bio-convergence Engineering, Korea University, Seoul 02841, Korea; jaeman0410@naver.com; 2 Proton Therapy Center, National Cancer Center, Goyang 10408, Korea; sblee@ncc.re.kr (S.B.L.); yklim@ncc.re.kr (Y.L.); kwancho@ncc.re.kr (K.C.); 3 Department of Medical Physics, Chinan Biomedical Technology Inc., Zhubei 30268, Taiwan; sungyong.park63@gmail.com

**Keywords:** fiber-optic, beam monitoring system, pencil beam scanning, proton therapy

## Abstract

This study describes the development of a beam monitoring system for the verification of entrance dose map in pencil beam scanning (PBS) proton therapy based on fiber optic radiation sensors (FORS) and the validation of this system through a feasibility study. The beam monitoring system consisted of 128 optical fibers optically coupled to photo-multiplier tubes. The performance of the beam monitoring system based on FORS was verified by comparing 2D dose maps of square-shaped fields of various sizes, which were obtained using conventional dosimeters such as MatriXX and EBT3 film, with those measured using FORS. The resulting full-width at half maximum and penumbra were compared for PBS proton beams, with a ≤2% difference between each value, indicating that measurements using the conventional dosimetric tool corresponded to measurements based on FORS. For irregularly-shaped fields, a comparison based on the gamma index between 2D dose maps obtained using MatriXX and EBT3 film and the 2D dose map measured by the FORS showed passing rates of 96.9 ± 1.3% and 96.2 ± 1.9%, respectively, confirming that FORS-based measurements for PBS proton therapy agreed well with those measured using the conventional dosimetric tools. These results demonstrate that the developed beam monitoring system based on FORS is good candidate for monitoring the entrance dose map in PBS proton therapy.

## 1. Introduction

**Figure 1:**
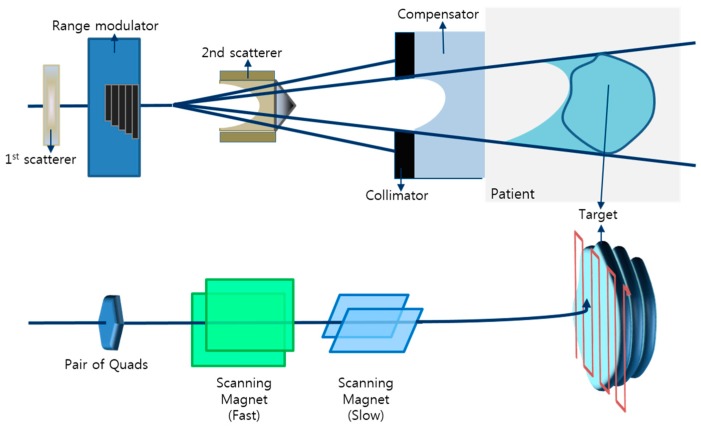
Proton beam delivery techniques: Passive scattering mode (**upper panel**) and Pencil beam scanning mode (**lower panel**).

Intensity modulated radiotherapy (IMRT), tomotherapy, and proton therapy are representative examples of novel radiotherapy methods, which can lead to better results when treating cancer than conventional 3D conformal radiotherapy [1,2,3]. In particular, proton therapy can treat cancer cells more effectively due to the characteristics of the Bragg peaks, which can deliver the maximum dose at the desired depth, rather than at a shallow depth [4,5,6]. Two types of beam delivery techniques are currently available for proton therapy: the passive scattering mode and pencil beam scanning (PBS). The passive scattering mode treats tumors in a desired block shape (tumor shape) using small-sized proton beams that scatter the protons, followed by spreading of the beams [7,8]. The PBS mode rapidly scans individually weighted spot beams without spreading small-sized proton beams and irradiates the tumors with the pencil beams. Because this method accurately controls the position and timing of proton beams, it needs sophisticated and precise treatment [9,10,11]. (Figure 1) The PBS mode uses weights and smaller beams than the passive scattering mode, delivering doses with the shape and size of the tumor. The treatment process of PBS mode involves diverse conditions, such as beam position, scanning velocity and the calculation of various complex factors, which results in planning that is less intuitive, all of which may lead to risk of radiation therapy accidents. To reduce the likelihood of this kinds of accidents, in X-ray therapy, beam monitoring systems were developed to monitor the beam delivered to the patient at the final stage of treatment (i.e., in the treatment room). Ion chamber-based integral quality monitoring (IQM, iRT Systems GmbH, Koblenz, Germany) and dolphin (Ion Beam Applications, Louvain-La-Neuve, Belgium) are representative commercially available beam monitoring systems for X-ray therapy. These systems are attached to a gantry and can measure irradiated beams in real-time during patient treatment. Subsequently, these systems determine if the actual irradiated dose is equal to the dose calculated by the treatment planning system (TPS) [12,13,14,15,16]. These systems, however, are expensive and have been developed for X-ray therapy only.

Although the MatriXX 2D ion chamber array (Ion Beam Applications) can be used as a dosimetric tool to measure PBS beams, this system is currently used for quality assurance only. Its thickness, however, makes it unsuitable for use as a beam monitoring system in the treatment room, because it is not easy to attach this device to a proton gantry. Moreover, the center-to-center distance between the ion chambers in the MatriXX is 7.62 mm, making it difficult to precisely and accurately measure PBS 3–4 mm proton beams [17]. Although various new dosimetric tools, including glass dosimeters, films, and TLDs, are both small and thin, they have several disadvantages, including their inability to perform real-time measurements and difficulty in accurately measuring the proton beam from a dosimetric perspective [18,19,20]. Although built-in ion chambers were already being used to verify the dose and position in proton therapy, the resolution of built-in equipment is not good enough to verify the entrance dose map. Therefore, new dosimetric tools are needed for use as beam monitoring systems in proton therapy. 

Recent studies have suggested the potential of optical fiber for proton dosimetry [21,22,23,24,25]. Fiber optic radiation sensor (FORS) based on fluorescent light were recently shown to be useful for proton dosimetry because, unlike scintillating fibers, they have no quenching effect. Although there was argument about the physical origins of visible light measured by optical fiber during proton irradiation, it is plausible that the visible light signal, which is proportional to the absorbed dose of proton beam, is not coming from the Cerenkov radiation but from the fluorescence light of the fiber. Darafsheh et al. reported that the signal in fiber optic dosimetry of proton beams is the fluorescence light of the fiber based on the spectral analysis obtained from bare fiber during proton beam irradiation [21]. The previous study also suggests that FORS is independent about energy and dose rate by revealing a constant signal value (or light yield) for various energy and dose rate. In addition, the fluorescence light is linearly related to the irradiated dose in the proton beam [25]. This study describes our development of a FORS based the beam monitoring system for the verification of entrance dose map, involving optical fibers in array forms, and an evaluation of the suitability of FORS as a beam monitoring system for PBS proton beams by comparing this newly developed system with the conventional dosimetric system for PBS proton beams. 

## 2. Materials and Methods 

### 2.1. System Configuration

**Figure 2:**
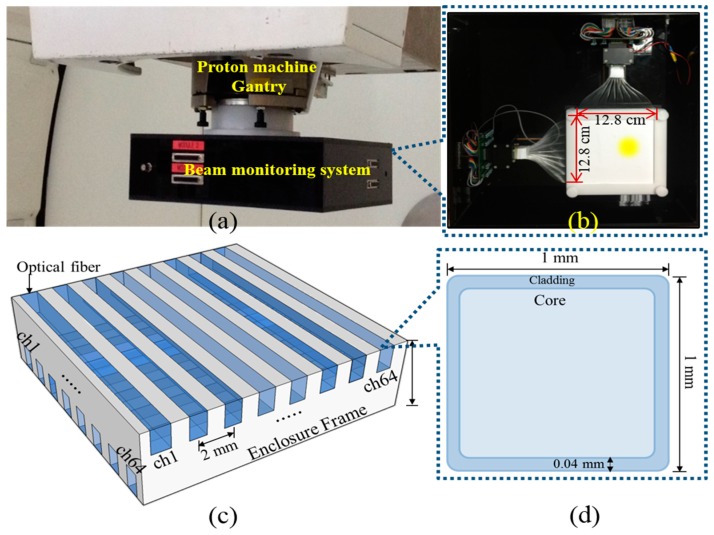
(**a**) Configuration of the developed FORS based proton beam dosimetry system; (**b**) The inside view of the developed proton beam dosimetry system; (**c**) Schematic image of the side-view of the FORS; (**d**) Schematic image of cross-section of the optical fiber.

The two-dimensional beam monitoring system was based on FORS (see Figure 2). The plastic optical fibers (POF: BCF-98, SAINT-GOBAIN CRYSTALS, Nemours, France) used in this system were square in shape, 1 mm in size, and transmitted only fluorescent light to photomultiplier tube (PMT). The POFs chosen in this study are step index optical fibers with a core/cladding structure. The outer length of the square shaped POFs is 1.0 mm and the cladding thickness is 0.04 mm, and the core thickness is 0.96 mm. The core of the POFs was a polystyrene material of refractive index n_core_ = 1.60. Plastic optical fibers (an acrylic material with refractive index n_clad_ = 1.49) were used as cladding. Each FORS consisted of two layers of optical fibers inserted into an enclosure frame made of acrylic materials of 3 mm total thickness. 128 optical fibers (64 fibers for x-coordinate, 64 fibers for y-coordinate) were arranged in the acrylic enclosure with center-to-center spacing of 2 mm (i.e., the edge-to-edge distance is 1 mm). The first layer was composed of 64 POFs arranged along the x-coordinate, and the second layer of 64 POFs arranged along the y-coordinate; thus, the same coordinate was rotated 90°. Although one dose plane is ~1 mm below the other, it seems to be OK to verify the entrance dose map at the treatment room. In our experiment, the optical fiber array was always installed in the plane perpendicular to the beam direction. 

The POFs of the layers were optically coupled to the photomultiplier tubes (PMTs) of each channel (H12428-203, Hamamatsu Photonics, Hamamatsu, Japan). Each channel of PMT is connected to the electronic circuit which is designed to convert current signal from PMT into the voltage. The voltage generated in electronic circuit was amplified using pre-amp and transmitted to the NI-DAQ system (National Instruments, Austin, TX, USA) through the D-sub cable. Signals were obtained through the NI-DAQ 9188 and NI-9205 systems using self-developed software. 

In our experimental system, PMT signals were processed using a data acquisition system with a 1 kHz acquisition frequency, meaning that one 2D-data set from 128 channels was acquired every 1 ms. Based on the fact that the beam current at nozzle is ~5 nA for maximum energy in PBS mode, the developed system can measure the dose with dose rate up to ~0.2 MU/ms. Therefore, if the dose rate is higher than this limit, the individual spots cannot be resolved and the higher speed DAQ system should be applied. To reduce the optical crosstalk, the optical fiber was inserted in “enclosure frame” and firmly attached to PMT tube. And then, noise signal from optical crosstalk was eliminated based on the comparison with reference measurement (i.e., ion chamber measurement).

### 2.2. PBS Beam Measurement

**Figure 3:**
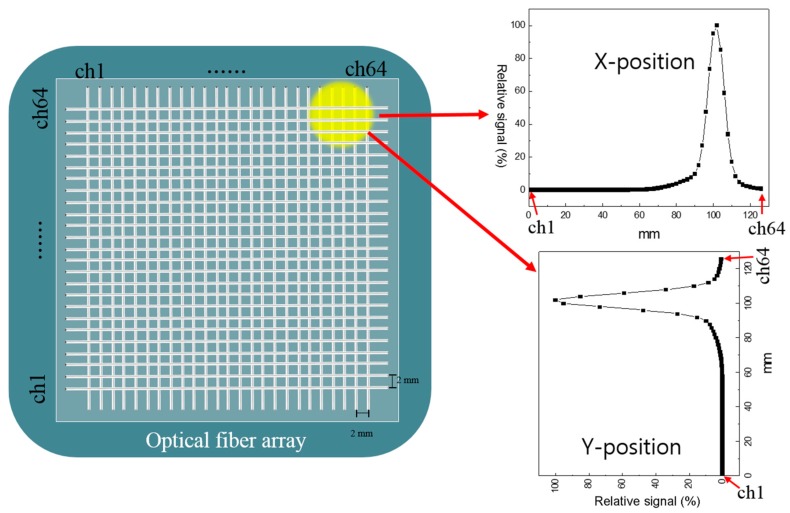
Schematic image of position localization using the FORS.

The beam monitoring system based on FORS was evaluated by obtaining the spot profile created by irradiating the beams of a square-shaped field on the FORS. Gaussian fitting was applied to determine the center (beam position) of the PBS proton beams. The measurement using high resolution detector showed that PBS beam has a Gaussian shape with different width for different energy. Because optical fiber array has a limited resolution (i.e., 2 mm), one had to fit the measured value to make the dose map. The measured Gaussian beam shape of proton pencil beam, which was also inserted to TPS, was used to fit our data. As seen in Figure 3, several fibers record a signal and each fiber corresponds to each x or y-coordinates. The signal of proton beam in each optical fiber is proportional to the length exposed to proton beam. The results of this system were compared with data obtained using MatriXX and EBT3 film, both of which are conventional dosimetric tools. Square PBS beams of different sizes were irradiated, and high energy (227.1 MeV) and low energy (122.6 MeV) beams were irradiated to determine the effect of irradiation on energy. 

Five irregularly-shaped fields of various sizes were irradiated with PBS beams. Gaussian fitting was applied to the measured profiles of these irregularly-shaped fields for two energies (122.6 and 227.1 MeV), resulting in the center value for the PBS beam. The beam modeling value of the TPS was applied to the center value. As in the irradiation of square-shaped fields, 2D dose distribution obtained using FORS was compared with the data measured by MatriXX and EBT3 films. 

### 2.3. Comparison Methods

To verify the performance of FORS, its determinations of full width half maximum (FWHM: 50–50%) and penumbra (20–80%) at the isocenter position were compared with those measured by MatriXX and EBT3 film. Doses to an irregularly-shaped field were determined using the gamma evaluation method, a quantitative 2D dose verification method [26]. The gamma evaluation method considers both the dose difference (DD) and distance-to-agreement (DTA) and confirms whether these tolerances are satisfied for each measured point. This method has been shown to detect a 3% DD in low-depth gradient areas and 3 mm DTA in high-depth gradient areas, making it sensitive to both components. A gamma index (GI) of less than 1, which corresponds to DD < 3% and DTA < 3 mm, represents good agreement between the values calculated by TPS and those measured by each dosimetry tool. GI was evaluated using commercially available Omnipro IMRT software for MatriXX measurements and commercially available RIT113 software for EBT3 film and FORS measurements. 

## 3. Results

**Figure 4:**
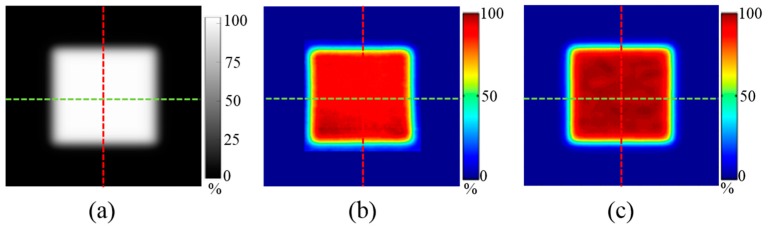
An example of measured 2D dose distributions using various dosimetric tools (Case 3 of the square-shaped field in Table 1). (**a**) MatriXX; (**b**) EBT3 film; (**c**) FORS.

Figure 4 shows a 2D dose distribution of square-shaped PBS beams (proton energy: 122.6, 227.1 MeV) measured using MatriXX, EBT3 film, and FORS. Table 1 shows the FWHM and penumbra (20–80%) values on horizontal and vertical lines in the 2D dose distributions measured by MatriXX, EBT3 film, and FORS, as well as differences between the values measured using both MatriXX and EBT3 film and those measured using FORS. The differences in FWHM values (i.e., the width between 50% of the maximum dose) between MatriXX and FORS based values and between EBT3 film and FORS based measurements were 1.2 ± 0.5% (range, 0.4–1.8%) and 0.8 ± 0.6% (range, 0.2–1.6%), respectively (Table 1). The differences in the penumbra values (the width of 20–80% of the maximum dose) between the MatriXX and FORS based values and between the EBT3 film and FORS based measurements were 1.3 ± 0.5% (range, 0.3–2.0%) and 1.5 ± 0.5% (range, 0.0–2.0%), respectively (Table 1). The relationships between FWHM and penumbra with regard to various sizes of square-shaped fields show that the FORS system performed similarly to the conventional dosimetric tools. 

**Figure 5:**
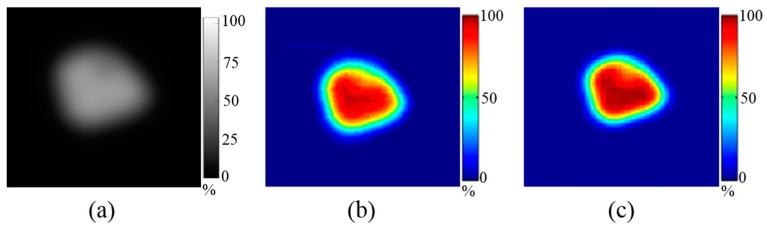
An example of measured 2D dose distribution using various dosimetric tools for the irregular-shaped field. (Field 4 in Table 2) (**a**) MatriXX; (**b**) EBT3 film; (**c**) FORS.

**Figure 6:**
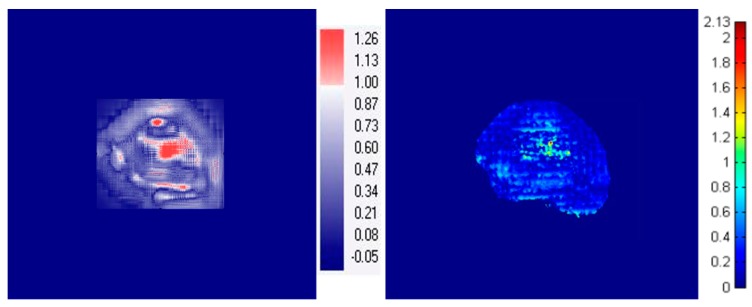

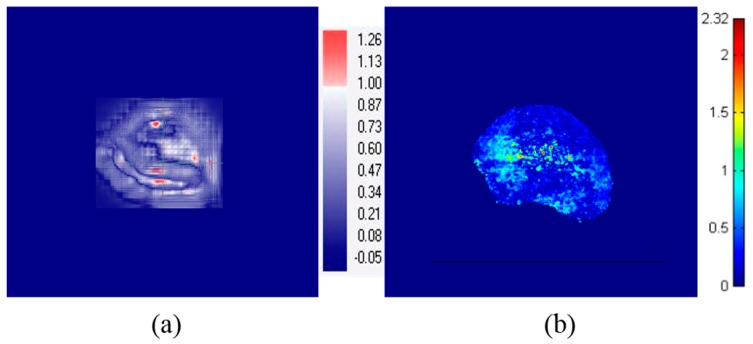
Example of dose comparison using the gamma evaluation method for an irregularly-shaped field. (Field 5 in Table 2). (**a**) MatriXX vs. FOR; (**b**) EBT3 film vs. FORS. The number denotes the gamma index.

Figure 5 shows an example of 2D dose distribution for PBS beams (energy: 122.6, 227.1 MeV) from irregularly-shaped field 4 in Table 2, which had been measured using MatriXX, EBT3 film and FORS. The GI passing rates (3%, 3 mm threshold level: 30%) of the 2D dose maps obtained using the values obtained for MatriXX and FORS were 96.8 ± 1.1% for the 122.6 MeV PBS beam and 97.0 ± 1.4% for the 227.1 MeV PBS beam. Similarly, the GI passing rates of the FORS relative to the EBT3 film measured values were 96.7 ± 1.0% for the 122.6 MeV PBS beam and 95.7 ± 2.3% for the 227.1 MeV PBS beam. Figure 6 shows a gamma image result obtained by applying the gamma evaluation method to FORS measured values relative to the MatriXX and EBT3 film measured values of irregularly-shaped field 5 in Table 2.

## 4. Discussion

The findings presented here indicate that a beam monitoring system based on fluorescence light can simultaneously verify the 2D dose distribution, including FWHM, penumbra, and gamma evaluation, in patients undergoing PBS proton therapy. The differences among patients treated with MatriXX, EBT3 Film, and FORS plans for the FWHM, penumbra, and failure rate in gamma index were small, indicating that the 2D dose distributions calculated using FORS were in good agreement with conventional dosimetric tools.

Beam monitoring is important in PBS proton therapy, because the dose map will change if some beams do not irradiate the planned position. A patient-specific QA method (pre-treatment QA) is used to validate the 2D dose distribution by comparing it with the calculated dose distribution obtained from the TPS. Although this method can detect systematic errors that occur during beam irradiation, it cannot measure the beams during patient treatment; rather it measures the beam during QA. However, measurement of pre-treatment QA cannot detect any unexpected random errors that result in irradiation with unwanted beams. One of the main advantages of the beam monitoring system based on FORS is its ability to evaluate beam delivery in real-time while a patient is being treated. This requires the dosimetric system to be physically thin and cause minimal proton beam interference, as the system should be located between the proton gantry and the patient. The system described in this report is therefore very promising, as it can be easily attached to the gantry, enabling proton beams to be measured during treatment without a large interference. 

Although the FORS induced perturbation can be small, it is important to know how much of an influence the device has on the spot size, particularly for low energy. To examine the perturbation due to the device, the spot sizes (one sigma) were measured for various energies with/without the device (i.e., FORS) in air at isocenter with EBT3 film. Compared to original spot sizes, the increased spot sizes due to the device ranged from 0.20 mm to 0.28 mm for various energies (E = 102 MeV to 197 MeV). For low beam energy (E = 102.7 MeV) and very low beam energy (E = 102.7 MeV with 7cm range shifter), the increased spot sizes due to the device were 0.24 mm and 0.23 mm, respectively, which mean ~2% of increase in spot sizes. These results suggest that the increase in spot size due to FORS device is not significant compared to original spot size. To use the device during treatment, however, the perturbation should be resolved by taking the matter into account in the treatment planning stage. For example, although the range shifter or compensator in proton therapy can also cause the perturbation of incoming proton beam, the treatment planning system can be commissioned to include the perturbation from this device.

There are some limits in current study. First, the 2D dose maps from clinical cases should be compared to dose maps measured with developed FORS system. Second, high-speed DAQ system should be applied to resolve the high speed individual proton beam spots.

## 5. Conclusions

The results of this study suggest that the developed system, which addresses the drawbacks of conventional systems, has good real-time monitoring capabilities for pencil beam scanning proton therapy. Because it can be easily attached to a proton gantry without large beam interference, it may be useful to verify the entrance dose map in the treatment room.

## Figures and Tables

**Table 1:** Profiles for square-shaped fields, including FWHM and penumbra, using MatriXX, EBT3 film, and FORS.

			Horizontal Profile (x-Axis)					Vertical Profile (y-Axis)				
	Energy		Measured Data (mm)			%Diff.		Measured Data (mm)			%Diff.	
		Case	MatriXX	EBT3 Film	FORS	MatriXX vs. FORS	EBT3 Film vs. FORS	MatriXX	EBT3 Film	FORS	MatriXX vs. FORS	EBT3 Film vs. FORS
FWHM (mm)	122.6 MeV	#1	44.3	44.8	45.1	1.8	0.6	44.3	44.4	45.0	1.7	1.3
		#2	63.8	64.0	64.9	1.8	1.4	65.0	64.1	64.2	1.1	0.2
		#3	104.8	103.7	103.9	0.9	0.2	104.4	104.4	106.0	1.5	1.5
	227.1 MeV	#1	43.6	44.1	44.2	1.4	0.2	44.1	43.9	44.6	1.1	1.6
		#2	66.4	66.4	65.3	1.7	1.6	63.4	63.8	64.1	1.2	0.5
		#3	103.6	104.7	104.1	0.5	0.5	104.0	104.0	104.4	0.4	0.3
Penumbra (mm)	122.6 MeV	#1	16.2	16.0	16.2	0.4	1.3	16.3	15.7	16.0	1.5	2.0
		#2	16.9	16.7	16.7	1.5	0.0	16.5	16.0	16.3	1.5	1.9
		#3	16.8	16.3	16.5	1.5	1.4	16.9	16.4	16.7	1.4	2.0
	227.1 MeV	#1	9.8	9.5	9.6	1.6	1.8	10.0	9.7	9.8	2.0	1.1
		#2	9.7	9.4	9.5	1.8	1.5	9.9	9.6	9.8	0.9	1.8
		#3	9.7	9.5	9.7	0.3	1.8	10.0	9.8	9.9	1.0	1.5

**Table 2:** Mean passing rates in gamma indices based on the gamma evaluation method for irregularly-shaped fields using MatriXX, EBT3 film, and FORS (units: %).

Energy	Tool	Field 1	Field 2	Field 3	Field 4	Field 5	Mean
122.6 MeV	MatriXX vs. FORS	98.4	97.1	96.4	97.1	95.1	96.8 ± 1.1
	EBT3 Film vs. FORS	97.4	95.7	95.3	97.6	97.3	96.7 ± 1.0
227.1 MeV	MatriXX vs. FORS	96.2	99.6	96.2	97.5	95.7	97.0 ± 1.4
	EBT3 Film vs. FORS	96.9	96.5	91.1	96.3	97.7	95.7 ± 2.3
